# Miscellany

**Published:** 1906-07

**Authors:** 


### MISCELLANY.

The United States Civil Service Commission announces examinations July 5 and 6, 1906, for a medical interne at the government hospital for the insane; also, for a hospital interne on the Isthmus of Panama. These examinations will be held at the usual places.

The meeting of the Military Surgeons Association to be held in Buffalo, September 11-14, 1906, will be an event of great interest and will attract many visitors to the city. On such occasions the question of hotel accommodations is an important one. The leading hotels here are prepared to furnish accommodations of excellent quality. The Iroquois, the Touraine, the Lennox, the Broezel and the Stafford are all excellent hostelries, differing in location and price, and we advise those interested to secure accommodations in one or the other of them at an early date. In our advertising columns will be found cards giving further information.

